# Physical activity and screen time behavior, and non-alcoholic beverage consumption during the COVID-19 pandemic in the longitudinal study of adult health (ELSA-Brasil)

**DOI:** 10.3389/fnut.2025.1503010

**Published:** 2025-05-02

**Authors:** Yazareni José Mercadante Urquía, Haysla Xavier Martins, Taísa Sabrina Silva Pereira, Letícia Batista de Azevedo, Luís Carlos Lopes Júnior, Maria Del Carmen Bisi Molina

**Affiliations:** ^1^Post-graduate Program in Collective Health, Universidade Federal do Espírito Santo, Vitória, Brazil; ^2^Department of Health Sciences, Universidad de las Américas Puebla, Puebla, Mexico; ^3^Post-graduate Program in Nutrition and Health, Universidade Federal do Espírito Santo, Vitória, Brazil; ^4^Post-graduate Program in Nutrition and Longevity, Universidade Federal de Alfenas, Alfenas, Brazil

**Keywords:** screen time (ST), physical activity, carbonated beverages, sugar-sweetened beverages (SSB), COVID-19, health behavior, lifestyle

## Abstract

**Background:**

The COVID-19 pandemic affected various dietary and lifestyle behaviors. Understanding the relationship between physical activity, screen time, and beverage consumption is critical to identify at-risk populations, particularly individuals with chronic non-communicable diseases (NCDs), for targeted intervention strategies.

**Objective:**

This study aimed to evaluate the relationship between physical activity, screen time behavior patterns, and non-alcoholic beverage consumption among participants in ELSA-Brasil during the COVID supplementary study.

**Methods:**

The study was conducted in five of the six ELSA-Brasil research centers, from July 2020 to March 2021. Sociodemographic variables, physical activity, screen time, non-alcoholic beverage consumption, and lifestyle factors were analyzed using bivariate analyses and binary logistic regression models.

**Results:**

The total sample consisted of 4,442 participants with a mean age of 62.0 ± 8.6 years. Significant gender differences were found in sociodemographic and lifestyle variables, influencing beverage consumption patterns. Participants with excessive screen time and physical inactivity were more likely to consume sugar-sweetened beverages. Specifically, men exhibited a significant association with an odds ratio of 2.44 (95%CI: 1.54–3.84), while women had an odds ratio of 1.72 (95% CI: 1.02–2.91). Men with appropriate screen time but physically inactive behavior had nearly double the likelihood of consuming sugar-sweetened artificial beverages. Additionally, men with excessive screen time and physical inactivity had higher consumption of industrialized juices (OR: 1.93; 95% CI: 1.22–3.06), while women were more likely to consume soft drinks (OR: 2.28; 95%CI 1.10–4.72).

**Conclusion:**

These findings underscore the importance of addressing both screen time and physical activity in public health initiatives, through gender-specific approaches that account for socioeconomic disparities when prioritizing interventions.

## 1 Introduction

The social restriction measures resulting from the COVID-19 pandemic have led to modifications in various aspects of life across different populations (1), with significant distinctions based on pre-existing health and socioeconomic conditions (2). Among the changes observed, there was a substantial increase in the time spent on screen devices, such as smartphones, video games, computers, and televisions (3), and, conversely, a reduction in physical activity (4). In Brazil, a cross-sectional study conducted by Malta et al. (5) evidenced an increase in screen time during the COVID-19 pandemic compared to the pre-pandemic period, with an average increase of 1 h and 45 min for TV and 1 h and 30 min for computer or tablet use. Regarding physical inactivity, a 26% increase was observed in Brazilian adults (6). Moreover, in a sex-stratified analysis using data from the Brazilian longitudinal study of adult health (ELSA-Brasil), Molina et al. (7) observed a reduction in physical activity levels during the COVID-19 pandemic, with declines of 195.5 (standard deviation [SD] ± 1,146.4) and 240.5 (SD ± 1,474.2) metabolic equivalents/week among women and men, respectively.

Social distancing measures also significantly impacted dietary consumption (8, 9). A study conducted with adults from southeastern Brazil evidenced an increase in snacking between meals, higher consumption of morning snacks, and a decrease in breakfast consumption (10). These changes are partly attributed to the repercussions of the pandemic on individuals’ mental health (9, 11), where coping with feelings such as anxiety or even boredom often involves eating (12, 13).

Regarding the consumption of non-alcoholic beverages during the pandemic, a cross-sectional study with 4,022 Australian adults identified that most did not change their consumption of sugary drinks. Among those who increased their frequency of intake, the most likely were men and those with children at home, while those with higher education levels showed a reduction in frequency (14). These results align with those of Park and collaborators (2023) in a sample of 4,034 adults from the United States, where being male, having lower education, and having children were associated with a higher likelihood of being a high consumer (≥2 times/day) of sugary drinks (15). A general increase in coffee consumption was observed during the pandemic (16, 17), with remote work being identified as a driver of this global demand increase (18).

A comprehensive understanding of the relationship between physical activity, screen time, and non-alcoholic beverage consumption is critical, as these represent modifiable risk factors for non-communicable diseases (NCDs). Their interplay during the pandemic may have exacerbated health vulnerabilities and disparities. The COVID-19 pandemic created a unique opportunity to examine how abrupt lifestyle changes influenced health behaviors.

By examining these relationships in ELSA-Brasil participants – a cohort with detailed pre-pandemic data – we can isolate pandemic-specific effects and identify high-risk groups requiring targeted interventions. Although the COVID supplementary study is cross-sectional, the availability of baseline data from ELSA-Brasil enables a more nuanced interpretation of pandemic-related behavioral changes. This investigation underscores the urgency of examining these combined behaviors to mitigate long-term health consequences. The ELSA-Brasil cohort, with its robust pre-pandemic data and diverse adult population, provides an exceptional platform to assess these relationships with greater validity than conventional cross-sectional studies, offering insights particularly valuable for public health strategies targeting similar populations during future health crises.

Engaging in unhealthy behavior makes individuals susceptible to adopting other deleterious habits (16). Furthermore, the negative modifications resulting from COVID-19, such as physical inactivity (17), sedentary behavior – represented by increased screen time – (18), and excessive consumption of certain non-alcoholic beverages (19, 20), amplify the burden of NCDs. These changes contribute as risk factors for the occurrence of obesity and other NCDs. Therefore, it is essential to evaluate these lifestyle habits in a combined manner.

We hypothesized that lower physical activity levels and higher screen time would be associated with greater consumption of sugar-sweetened beverages during the pandemic period. This hypothesis was grounded in previous evidence showing that sedentary behaviors and unhealthy dietary patterns frequently co-occurred during the pandemic, as observed in Latin American populations (21) and Brazilian adolescents (22), while stress-related coping mechanisms during lockdowns drove parallel increases in both screen time (23, 24) and consumption of energy-dense foods like sugary drinks through emotional distress pathways (12, 13). The ELSA-Brasil design permitted examination of these associations while controlling for relevant confounders.

This study addresses critical gaps in understanding the interplay between physical activity, screen time, and non-alcoholic beverage consumption during the COVID-19 pandemic. By focusing on Brazilian public servants, both active and retired, it provides unique insights into pandemic-induced behavioral changes. The originality of this research lies in its comprehensive evaluation of these combined lifestyle factors and its detailed analysis by type of beverage, a novel approach not extensively explored in previous studies among adults. In the post-pandemic context, our study offers crucial empirical evidence on the relationships between these factors. The findings contribute to a broader understanding of the health risks associated with pandemic-induced lifestyle changes, with the potential to inform the development of targeted interventions for at-risk groups. These insights strengthen the theoretical foundation on the adverse health effects of such lifestyle changes in future similar scenarios.

Given the above, this study aims to evaluate the relationship between physical activity and screen time behavior patterns and the consumption of non-alcoholic beverages among public servants, both active and retired, participating in ELSA-Brasil during the COVID-19 pandemic.

## 2 Materials and methods

### 2.1 Study design and population

The present study is a cross-sectional quantitative analysis that utilizes data from a supplementary study conducted during the COVID-19 pandemic (2020–2021). This supplementary study is part of a larger multicenter cohort, ELSA-Brasil, which began its baseline period in 2008–2010 with 15,105 participants, both active and retired public servants, aged 35 to 74 at the time of recruitment. ELSA-Brasil aims to investigate associations between chronic diseases, primarily cardiovascular diseases and diabetes, and biological, behavioral, environmental, occupational, and social factors (25). The study is conducted at six research centers located in five public universities and one research institute of the Brazilian Ministry of Health (26). The supplementary study, named the COVID wave, was implemented to assess the pandemic’s impact on the health of the cohort participants. Both ELSA-Brasil and the supplementary study received approval from the Ethics Committees of the participating research centers, and all participants provided informed consent, ensuring data confidentiality and ethical treatment (27).

In the supplementary COVID study (2020–2021), participants from five of the six research centers were invited from three regions of the country, including the Northeast, South, and Southeast (Federal Universities of Bahia, Rio Grande do Sul, Espírito Santo and Minas Gerais; and Oswaldo Cruz Foundation [FIOCRUZ], located in Rio de Janeiro). A total of 5,544 individuals agreed to respond to the questionnaires administered during this phase of the study. Participants without data on screen time (*n* = 573), beverage consumption (*n* = 18), and physical activity (*n* = 511) were excluded from the analysis due to non-response. The total sample size included in the analysis was 4,442 participants. Supplementary Figure 1 illustrates the flowchart of cohort participants and their follow-up over the study periods.

Outliers were adjusted to the 99th percentile value of the respective variable (screen time > 16 h/day and physical activity > 645 min/week) to minimize the impact of extreme values on the analysis.

This study adhered to the Declaration of Helsinki guidelines and received approval from ELSA-Brasil and the Research Ethics Committees of all participating institutions, with the following approval numbers: 343/06 at the FIOCRUZ-RJ Institute (COVID Study – N° 4.063.982), 041/06 at UFES (COVID Study - N° 4.090.940), 186/06 at UFMG (COVID Study - N° 4.082.055), 194/06 at UFRGS (COVID Study - N° 4.023.601), and 027/06 at UFBA (COVID Study - N° 4.067.184).

### 2.2 Data collection and measures

Data were collected during the COVID wave, from July 2020 to March 2021, conducted online using a specific platform and through telephone contact due to social distancing measures implemented in various Brazilian states during this period. Sociodemographic, lifestyle, and food and beverage consumption data were collected.

#### 2.2.1 Sociodemographic data

From the data of Wave 3 (2017–2019) of ELSA-Brasil, the participants’ age was calculated by adding an average of 3 years, and the *per capita* income was treated as a continuous variable. Additionally, data collected during the COVID wave included sex (male or female), self-reported race/ethnicity (categorized into two categories: White, and Black, Brown, Yellow, and Indigenous), according to the classification of the *Instituto Brasileiro de Geografia e Estatística* (28); current employment status (active or retired), and whether participants worked remotely during the pandemic (yes or no).

#### 2.2.2 Exposure: physical activity and screen time behavior

To characterize physical activity and screen time behavior, a composite variable was created combining both physical activity behavior and screen time. In the COVID wave of the ELSA-Brasil study, physical activity (PA) was assessed using the short version of the International Physical Activity Questionnaire (IPAQ), adapted and validated for the Brazilian population (29). The questionnaire included the following questions: “Since the beginning of social distancing, on average how many days a week do you take walks in your free time?”; “Since the beginning of social distancing, how many days a week do you do vigorous physical activities in your free time?”; and “Since the beginning of social distancing, how many days a week do you do moderate physical activities?.” PA was reported in minutes per week by multiplying the weekly frequency by the duration of each activity. Only PA sessions lasting at least 10 consecutive minutes were considered. Vigorous PA was defined as activities requiring significant physical effort and causing much faster breathing than usual, while moderate PA required moderate effort and caused slightly faster breathing than usual. Participants were considered physically active if they engaged in ≥150 min/week of moderate-to-vigorous PA and physically inactive if they engaged in <150 min/week of moderate-to-vigorous PA (30).

Screen time was estimated from participants’ self-reported time spent in front of screen devices during social distancing, using the question “How much time are you now spending in front of any screen (including cell phone, computer, TV, laptops, or others)?.” The data were obtained in hours per day and categorized using the sample’s median screen time hours as the cutoff point, adjusted for outliers based on the 99th percentile (16 h/day), into two categories: ≤6 h/day (Appropriate Screen Time) and >6 h/day (Excessive Screen Time). The cutoff point was based on the median screen time of the study sample, due to the lack of consensus in the current literature regarding recommended screen time for adults. Additionally, this cutoff coincides with that used in another significant study on the Brazilian population, which considered screen time ≥6 h/day as high (31).

Based on these variables, a recategorization was performed, creating four categories of the combined Physical Activity and Screen Time Behavior variable: “Appropriate Screen Time/Physically Active”; “Appropriate Screen Time/Physically Inactive”; “Excessive Screen Time/Physically Active”; and “Excessive Screen Time/Physically Inactive.”

#### 2.2.3 Outcome: non-alcoholic beverage consumption on the previous day

Non-alcoholic beverage consumption was assessed through a 24-h dietary recall with the question “Look at this list of drinks and check all that you consumed yesterday (from when you woke up until you went to sleep),” and data were obtained on the consumption of soft drink or soda, industrialized juices (boxed, bottled, concentrated, or powdered), sugar-sweetened artificial beverages (soda + industrialized juices), and coffee with and without sugar. Participants indicated whether they consumed each of these beverages on the previous day. The 24-h dietary recall was applied twice during the COVID wave, with a minimum interval of 1 month between the first and second application.

#### 2.2.4 Lifestyle covariates

Data on smoking were collected with the question “Do you currently smoke cigarettes?” with three response options (Never smoked, Former smoker, and Current smoker). Alcohol consumption data were collected with the question “Since the beginning of social distancing, have you consumed any type of alcoholic beverage?” with a dichotomous response (Yes or No).

### 2.3 Statistical analysis

Analyses were stratified by sex due to the interaction in the association between exposure and outcome, as well as the different patterns of PA/screen time behavior and beverage consumption between men and women. For evaluating differences between groups, Student’s T-test and ANOVA (Supplementary Table 1) were applied for continuous variables, and the Chi-Square test was used for categorical variables. Bivariate analyses were performed to assess the association between beverage consumption and sociodemographic, lifestyle, and PA/screen time behavior variables. Binary logistic regression analysis was performed using an initial unadjusted model, which was subsequently adjusted based on a theoretical model with two hierarchical levels (32): adjusted model 1 included sociodemographic variables (age, *per capita* income, remote work), and adjusted model 2 included sociodemographic and lifestyle variables (smoking and alcohol consumption). Additionally, a test was conducted to rule out multicollinearity. Variables remained in the model if they reached a significance level of 20% in the bivariate analyses. Descriptive and exploratory analyses were performed using SPSS version 23.0 with a significance level of 5%.

## 3 Results

The total study sample consisted of 4,442 participants with a mean age of 62.0 ± 8.6 years. Significant differences were found between genders in relation to sociodemographic and lifestyle variables (Table 1). Most of the sample consisted of women (58.2%) and individuals who were working (59.1%), with appropriate screen time and physical inactivity (45.4%), non-smokers (65.5%), and alcohol consumers during the pandemic (62.0%). Additionally, men exhibited a higher average screen time (*p* = 0.026).

**Table 1:** Sociodemographic characteristics and lifestyle among participants of the COVID supplementary study in ELSA-Brasil by gender (2020–2021).

Variables	Total (*n* = 4,442)	Sex		*p* value^a^
		Males (*n* = 1857)	Females (*n* = 2,585)	
	*n* (%)	*n* (%)	*n* (%)	
Age (years) ^(*n* = 4,346)^	62.0 ± 8.6	62.1 ± 8.9	61.9 ± 8.4	0.504
Race ^(*n* = 4,318)^				
White	2,477 (57.4)	1,070 (59.1)	1,407 (56.1)	0.057
Black, Brown, Yellow, and Indigenous	1841 (42.6)	742 (40.9)	1,099 (43.9)	
*Per capita* income (US$ - monthly)	811,6 ± 587,4	786,4 ± 551,7	829,9 ± 611,3	0.016*
Employment status ^(*n* = 4,416)^				
Employed/working	2,612 (59.1)	1,269 (69.0)	1,343 (52.1)	<0.001*
Retired	1804 (40.9)	571 (31.0)	1,233 (47.9)	
Remote work ^(*n* = 2,608)^				
Yes	2022 (77.5)	997 (78.6)	1,025 (76.5)	0.205
No	586 (22.5)	271 (21.4)	315 (23.5)	
Screen time behavior				
Below the median (≤6 h/d)	2,494 (56.1)	1,024 (55.1)	1,470 (56.9)	0.257
Above the median (> 6 h/d)	1948 (43.9)	833 (44.9)	1,115 (43.1)	
Screen Time (hours)	6.6 ± 3.6	6.7 ± 3.6	6.6 ± 3.5	0.026*
Physical activity behavior				
Physically inactive (<150 min/week)	3,569 (80.3)	1,433 (77.2)	2,136 (82.6)	<0.001*
Physically active (≥150 min/week)	873 (19.7)	424 (22.8)	449 (17.4)	
Physical activity and screen time (ST) behavior				
Appropriate ST/Physically Active	478 (10.8)	233 (12.5)	245 (9.5)	<0.001*
Appropriate ST/Physically Inactive	2016 (45.4)	791 (42.6)	1,225 (47.4)	
Excessive ST/Physically Active	395 (8.9)	191 (10.3)	204 (7.9)	
Excessive ST/Physically Inactive	1,553 (35.0)	642 (34.6)	911 (35.2)	
Smoking				
Non-smoker	2,909 (65.5)	1,116 (60.1)	1793 (69.4)	<0.001*
Former smoker	1,251 (28.2)	617 (33.2)	634 (24.5)	
Current smoker	282 (6.3)	124 (6.7)	158 (6.1)	
Alcoholic beverage consumption				
Yes	2,756 (62.0)	1,324 (71.3)	1,432 (55.4)	<0.001*
No	1,686 (38.0)	533 (28.7)	1,153 (44.6)	

a Chi-square test for categorical variables and Student’s *t*-test for continuous variables.

^*^p values <0.05, highlighted in bold, were considered statistically significant.

Beverage consumption varied according to sociodemographic and lifestyle variables (Table 2). The majority of soft drink or soda consumers were women (*p* < 0.001), working (*p* = 0.003), non-smokers (*p* < 0.001), and working remotely during the pandemic (*p* = 0.012). They were also physically inactive (*p* < 0.001) and predominantly exhibited combinations of appropriate or inappropriate screen time with physical inactivity (*p* < 0.001). A similar pattern was observed among consumers of other types of sugar-sweetened artificial beverages.

**Table 2:** Sociodemographic characteristics and lifestyle among participants of the COVID supplementary study in ELSA-Brasil by beverage consumption (2020–2021).

Variables	Beverage consumption on the previous day									
	Soft drink or soda		Sugar-sweetened artificial beverages		Industrialized juices		Coffee with sugar/ sweetener		Coffee without sugar	
	Yes *n* (%)	No *n* (%)	Yes *n* (%)	No *n* (%)	Yes *n* (%)	No *n* (%)	Yes *n* (%)	No *n* (%)	Yes *n* (%)	No *n* (%)
Gender ^(*n* = 4,375)^										
Male	371 (47.1)	1,486 (40.7)	660 (47.1)	1,197 (39.4)	360 (48.2)	1,497 (40.5)	1,092 (42.3)	765 (41.1)	509 (39.5)	1,348 (42.7)
Female	417 (52.9)	2,168 (59.3)	741 (52.9)	1844 (60.6)	387 (51.8)	2,198 (59.5)	1,487 (57.7)	1,098 (58.9)	778 (60.5)	1807 (57.3)
*p* value^a^	*p = 0*.001*		*p* = <0.001*		*p* = <0.001*		*p* = 0.405		*p* = 0.052	
Age (years) ^(*n* = 4,357)^										
	60.9 ± 8.7	62.3 ± 8.6	61.2 ± 8.7	62.4 ± 8.5	61.5 ± 8.8	62.1 ± 8.6	62.5 ± 8.7	61.4 ± 8.4	62.0 ± 8.4	62.0 ± 8.7
*p* value^a^	*p* = <0.001*		*p* = <0.001*		*p* = 0.059		*p* = <0.001*		*p* = 0.770	
Race ^(*n* = 4,318)^										
White	444 (58.1)	2033 (57.2)	782 (57.5)	1,695 (57.3)	414 (56.9)	2063 (57.4)	1,308 (52.2)	1,169 (64.6)	856 (68.4)	1,621 (52.9)
Black, Brown, Yellow, and Indigenous	320 (41.9)	1,521 (42.8)	577 (42.5)	1,264 (42.7)	313 (43.1)	1,528 (42.6)	1,200 (47.8)	641 (35.4)	395 (31.6)	1,446 (47.1)
*p* value^a^	*p* = 0.658		*p* = 0.895		*p* = 0.805		*p* < 0.001*		*p* < 0.001*	
*Per capita* income (US$) ^(*n* = 4,346)^										
	739,6 ± 532,8	827,1 ± 597,4	766,5 ± 565,6	832,3 ± 596,0	791,0 ± 595,2	815,8 ± 585,8	715,7 ± 530,7	943,4 ± 634,2	1,003,2 ± 660,3	733,0 ± 535,3
*p* value^a^	*p* = <0.001*		*p* = 0.001*		*p* = 0.299		*p* < 0.001*		*p* < 0.001*	
Employment status ^(*n* = 4,416)^										
Employed/ Working	501 (63.8)	2,111 (58.1)	877 (62.8)	1735 (57.5)	462 (62.0)	2,150 (58.6)	1,430 (55.8)	1,182 (63.8)	802 (62.8)	1810 (57.7)
Retired	284 (36.2)	1,520 (41.9)	519 (28.8)	1,285 (42.5)	283 (38.0)	1,521 (41.4)	1,134 (44.2)	670 (36.2)	476 (37.2)	1,328 (42.3)
*p* value^a^	*p* = 0.003*		*p* = 0.001*		*p* = 0.086		*p* < 0.001*		*p* = 0.002*	
Remote work ^(*n* = 2,608)^										
Yes	367 (73.3)	1,655 (78.5)	643 (73.5)	1,379 (79.6)	339 (73.7)	1,683 (78.4)	1,022 (71.7)	1,000 (84.6)	705 (87.9)	1,317 (72.9)
No	134 (26.7)	452 (21.5)	232 (26.5)	354 (20.4)	121 (26.3)	465 (21.6)	404 (28.3)	182 (15.4)	97 (12.1)	489 (27.1)
*p* value^a^	*p* = 0.012*		*p* = 0.001*		*p = 0*.031*		*p* < 0.001*		*p* < 0.001*	
Screen time behavior										
Below the median (≤6 h/d)	402 (51.0)	2092 (57.3)	733 (52.3)	1761 (57.9)	394 (52.7)	2,100 (56.8)	1,559 (60.4)	935 (50.2)	645 (50.1)	1849 (58.6)
Above the median (> 6 h/d)	386 (49.0)	1,562 (42.7)	668 (47.7)	1,280 (42.1)	353 (47.3)	1,595 (43.2)	1,020 (39.6)	928 (49.8)	642 (49.9)	1,306 (41.4)
*p* value^a^	*p = 0*.002*		*p* < 0.001*		*p = 0*.043*		*p* < 0.001*		*p* < 0.001*	
Physical activity behavior										
Physically inactive (<150 min/week)	686 (87.1)	2,883 (78.9)	1,190 (84.9)	2,379 (78.2)	621 (83.1)	2,948 (79.8)	2,147 (83.2)	1,422 (76.3)	971 (75.4)	2,598 (82.3)
Physically active (≥150 min/week)	102 (12.9)	771 (21.1)	211 (15.1)	662 (21.8)	126 (16.9)	747 (20.2)	432 (16.8)	441 (23.7)	316 (24.6)	557 (17.7)
*p* value^a^	*p* < 0.001*		*p* < 0.001*		*p = 0*.038*		*p* < 0.001*		*p* < 0.001*	
Physical activity and screen time (ST) behavior										
Appropriate ST/Physically Active	50 (6.3)	428 (11.7)	105 (7.5)	373 (12.3)	64 (8.6)	414 (11.2)	252 (9.8)	226 (12.1)	167 (13.0)	311 (9.9)
Appropriate ST/Physically Inactive	352 (44.7)	1,664 (45.5)	628 (44.8)	1,388 (45.6)	330 (44.2)	1,686 (45.6)	1,307 (50.7)	709 (38.1)	478 (37.1)	1,538 (48.7)
Excessive ST/Physically Active	52 (6.6)	343 (9.4)	106 (7.6)	289 (9.5)	62 (8.3)	333 (9.0)	180 (7.0)	215 (11.5)	149 (11.6)	246 (7.8)
Excessive ST/Physically Inactive	334 (42.4)	1,219 (33.4)	562 (40.1)	991 (32.6)	291 (39.0)	1,262 (34.2)	840 (32.6)	713 (38.3)	493 (38.3)	1,060 (33.6)
*p* value^a^	*p* < 0.001*		*p* < 0.001*		*p = 0*.033*		*p* < 0.001*		*p* < 0.001*	
Smoking										
Non-smoker	460 (58.4)	2,449 (67.0)	850 (60.7)	2059 (67.7)	467 (62.5)	2,442 (66.1)	1,578 (61.2)	1,331 (71.4)	869 (67.5)	2040 (64.7)
Former smoker	241 (30.6)	1,010 (27.6)	419 (29.9)	832 (27.4)	221 (29.6)	1,030 (27.9)	798 (30.9)	453 (24.3)	359 (27.9)	892 (28.3)
Current	87 (11.0)	195 (5.3)	132 (9.4)	150 (4.9)	59 (7.9)	223 (6.0)	203 (7.9)	79 (4.2)	59 (4.6)	223 (7.1)
*p* value^a^	*p* < 0.001*		*p* < 0.001*		*p = 0*.072		*p* < 0.001*		*p* = 0.006*	
Alcoholic beverage consumption										
Yes	493 (62.9)	2,263 (61.9)	876 (62.5)	1880 (61.8)	456 (61.0)	2,300 (62.2)	1,511 (58.6)	1,245 (66.8)	935 (72.6)	1821 (57.7)
No	295 (37.4)	1,391 (38.1)	525 (37.5)	1,161 (38.2)	291 (39.0)	1,395 (37.8)	1,068 (41.4)	618 (33.2)	352 (27.4)	1,334 (42.3)
*p* value^a^	*p* = 0.746		*p* = 0.665		*p = 0*.563		*p* < 0.001*		*p* < 0.001*	

a Chi-square test for categorical variables and Student’s *t*-test for continuous variables.

^*^p values <0.05, highlighted in bold, were considered statistically significant.

Individuals who consumed coffee, both with and without sugar or sweeteners, were predominantly white (*p* < 0.001), employed, working remotely during the pandemic (*p* < 0.001), non-smokers, and alcohol consumers during the pandemic. Consumers of both types of beverages were mostly physically inactive (*p* < 0.001), and the combined behaviors of physical activity and screen time showed predominant patterns of “Appropriate screen time/Physically inactive” and “Excessive screen time/Physically inactive” (*p* < 0.001).

The mean per capita income was significantly lower among those consuming soft drinks or soda, sugar-sweetened artificial beverages, and coffee with sugar, and significantly higher among those consuming coffee without sugar (data not shown in the table).

**Figure 1:**
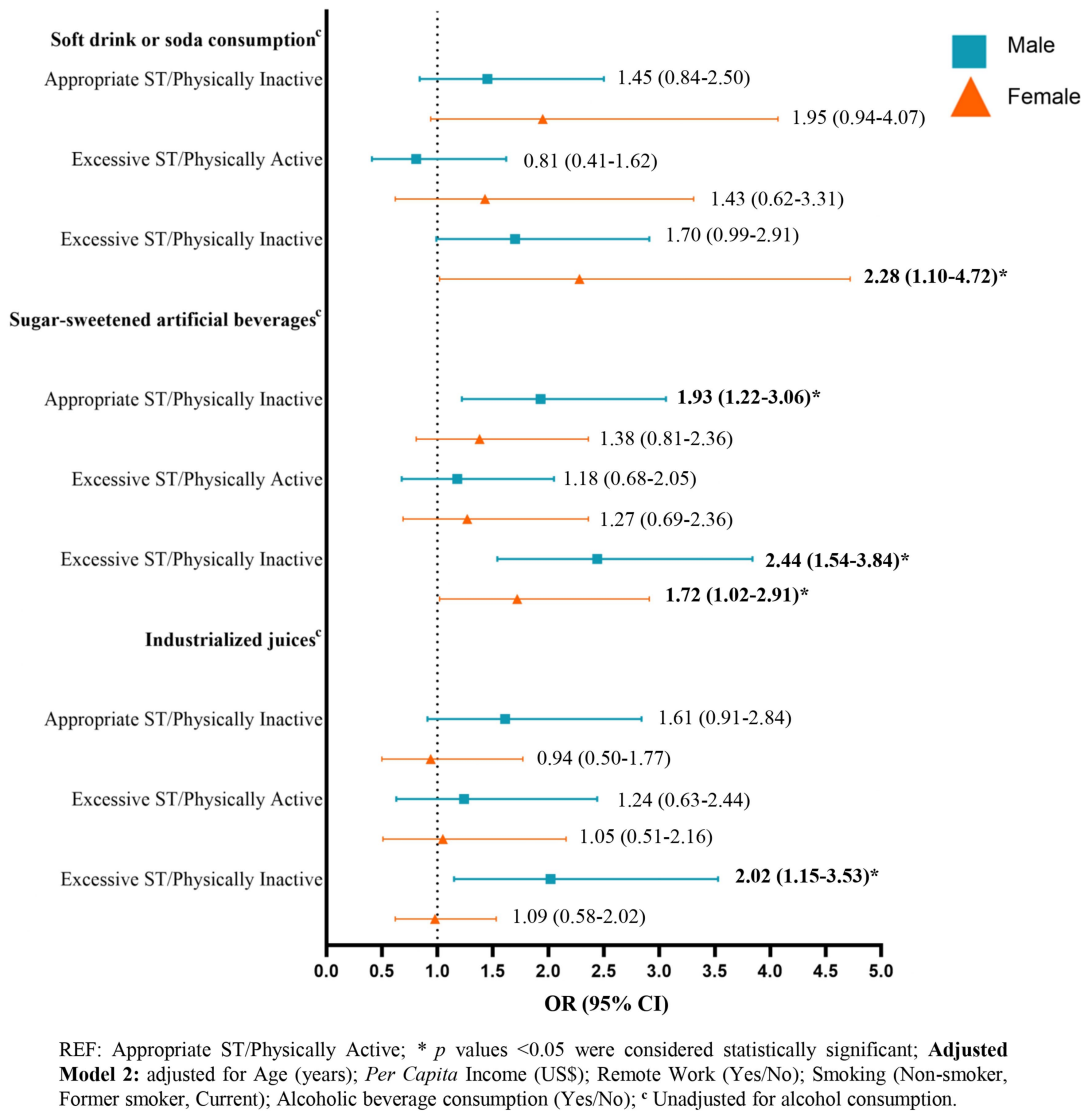
Adjusted logistic regression model: odds ratios (95% CI) for soft drinks, sugar-sweetened artificial beverages, and industrialized juices consumption by physical activity and screen time behavior in the ELSA-Brasil COVID-19 supplementary study (2020–2021).

The results of the binary logistic regression assessing the association between physical activity/screen time behavior and the consumption of non-alcoholic beverages are presented in Figure 1 and Supplementary Table 2. After adjusting for potentially confounding sociodemographic and lifestyle variables (Adjusted Model 2), it was identified that the behavior category of Excessive screen time/Physically inactive was associated with higher odds of consuming sugar-sweetened artificial beverages among both men and women.

Among men, the behavior of “Appropriate screen time/Physically inactive” was significantly associated with a 1.93-fold increase in the likelihood of consuming sugar-sweetened artificial beverages, whereas presenting a behavior of “Excessive screen time/Physically inactive” increased the likelihood by 2.44 times among men (95% CI 1.54–3.84) and 1.72 times among women (95% CI 1.02–2.91). Additionally, among men, the behavior of “Excessive screen time/Physically inactive” also increased the likelihood of consuming industrialized juices by 2.02 times (95% CI 1.15–3.53), and among women, it increased the likelihood of consuming soft drinks or soda by 2.28 times (95% CI 1.10–4.72). No associations were found between physical activity and screen time behavior and the consumption of coffee with or without sugar.

## 4 Discussion

In this study, the “Excessive Screen Time/Physically Inactive” behavior pattern during the COVID-19 pandemic among participants of the ELSA-Brasil cohort was associated with a 144% increase in the odds of consuming sugar-sweetened artificial beverages among men and a 72% increase among women. This pattern also correlated with a 128% higher probability of soda consumption in women and a 102% higher likelihood of consuming industrialized juices in men. Additionally, the “Adequate Screen Time/Physically Inactive” pattern increased the likelihood of consuming sugar-sweetened artificial beverages by 93% among men.

Despite the fact that no associations were found in the present study between physical activity/screen time behavior and the consumption of coffee with or without sugar, similar associations between *per capita* income and coffee consumption patterns identified in the present study have been found in previous studies conducted in the USA (33) and in Brazilian studies (34, 35). These studies found that individuals with higher income levels tended to consume less sweetened coffee (35). Additionally, as in the present study, the consumption of sugary beverages was also associated with lower *per capita* income among Brazilian adults (34). This can be explained by the lower cost per calorie of low-nutrient foods often chosen by individuals with lower *per capita* income, while those from higher socioeconomic backgrounds have greater nutritional knowledge and prefer healthier food choices (36).

These findings align with the existing literature highlighting the negative impacts of excessive screen time and physical inactivity, especially during periods of social restriction such as the COVID-19 pandemic (37). Excessive screen time has been correlated with higher consumption of unhealthy foods and beverages among adults, children, and adolescents in Brazil and other countries worldwide (21, 22, 38, 39).

Several physiological and behavioral pathways explain this association. Prolonged screen time promotes sedentary behavior, which disrupts glucose metabolism and increases cravings for high-calorie foods, including sugar-sweetened beverages (40). Concurrently, excessive screen exposure elevates stress and anxiety, triggering emotional eating as a coping mechanism, often manifesting as a preference for sugary beverages (41). These mechanisms could synergistically exacerbate sugar-sweetened beverages consumption during pandemic-related restrictions.

The patterns observed in the present study reflect the impact of screen time and physical inactivity on beverage consumption behaviors. During the COVID-19 pandemic, imposed restrictions led to increased time spent at home and remote work, contributing to increased screen time and sedentary behaviors (23). Even before the pandemic, a study analyzing screen time trends during leisure time among Brazilian adults from 2016 to 2021 found that the average time spent on devices such as mobile phones, computers, and tablets increased significantly, while TV viewing time remained stable (42).

Furthermore, during the pandemic, a cross-sectional study with ELSA-Brasil participants on remote work showed that working from home was associated with a significant increase in screen time and cumulative sitting time of more than 8 h per day, as well as higher domestic physical activity but not leisure-time physical activity (43). These results reflect the impact of lockdown measures on reducing physical activity and increasing sedentary behavior. Similarly, another study with ELSA-Brasil participants indicated that individuals with diabetes experienced a significant increase in screen time (1.3 h/day) and sitting or reclining time (1.4 h/day) during the pandemic, while physical activity decreased notably (44).

Several mechanisms may explain the observed associations. Screen time often displaces physical activity, leading to prolonged periods of sedentary behavior, which is independently associated with poor dietary habits, including increased consumption of sugary drinks (45). Additionally, a study with young Colombian adults observed a mild negative correlation between daily physical activity and the frequency of sugary drink consumption, particularly in men, with higher weekly vigorous physical activity being associated with lower intake of these drinks (46). Multiple mechanisms have been suggested to explain the relationship between screen time and unhealthy food consumption, including exposure to advertising and “mindless eating” (38, 47). A sedentary lifestyle, combined with frequent consumption of sugary drinks, creates a synergistic effect, contributing to adverse health outcomes (48–51). Studies have shown that physical inactivity can influence the consumption of sugary drinks (46, 52).

Moreover, stress and the disruption of normal routines during the pandemic may have intensified these behaviors: increased consumption of unhealthy foods, physical inactivity, and sedentary behavior (23). A systematic review identified physical inactivity and increased screen time as significant factors associated with weight gain during the COVID-19 pandemic (23).

In this context, the pandemic has been associated with increased screen time as a coping mechanism, which in turn can lead to higher consumption of unhealthy foods and sugary drinks (23, 24). The relationship between physical inactivity and sugary drink consumption may be mediated by brain reward responses to food cues, which are inversely associated with physical activity (53, 54), as well as the availability of time and ease of access to these drinks during sedentary activities that increased during the pandemic.

In pre-pandemic studies with participants from the ELSA-Brasil Wave 1, it was identified that most individuals engaged in low physical activity (76.9%), similar to the current study where most were physically inactive (80.3%) (55). Additionally, in the baseline (Wave 1) of the study, those who consumed sweetened beverages predominantly had a low level of physical activity, while those who consumed unsweetened and artificially sweetened beverages generally participated in moderate to intense physical activity. Furthermore, in that wave, individuals with a normal nutritional status reported higher consumption of sugary drinks, while those with obesity consumed more sugar-sweetened artificial beverages (55).

A study conducted with adults and adolescents from eight Latin American countries, including Brazil, found that 30.49% of the sample exhibited a combination of unhealthy behaviors: spending a lot of time in front of a screen and consuming sugary drinks (21). In Brazil, Chile, Peru, and Ecuador, the combination of an unhealthy diet or sugary drink consumption along with excessive screen use predominated (21). The authors concluded that these unhealthy behaviors were highly prevalent, highlighting the association between sedentary behavior and sugary drink consumption (21).

The WHO defines physical activity as any bodily movement produced by skeletal muscles which requires energy expenditure. Sedentary screen time refers to the time spent viewing screen-based entertainment (TV, computer, mobile devices), excluding screen-based games that require physical activity or movement (30). Screen time is a proxy variable for sedentary behavior. There is robust evidence establishing a relationship between sedentary behavior and all-cause mortality, as well as cardiovascular diseases, type 2 diabetes, and metabolic syndrome. Additionally, there is moderate evidence suggesting an association with the incidence rates of ovarian, colon, and endometrial cancers (48). A Brazilian cohort study identified a higher risk of mortality among adults who spend more time watching television, regardless of physical activity and other variables (49).

On the other hand, research indicates that high consumption of sugary drinks is linked to adverse metabolic outcomes, including an increased risk of obesity, type 2 diabetes, and metabolic syndrome (50, 51, 56). Due to their ability to rapidly and significantly raise blood glucose and insulin levels, sugary drinks contribute to a high dietary glycemic load. Consuming a diet with a high glycemic load is known to promote glucose intolerance and insulin resistance, as well as increased fat accumulation, particularly in the visceral regions, exacerbating the risk of metabolic diseases (50). Within the ELSA-Brasil cohort in Waves 1 and 2, higher consumption of sugary drinks was found to significantly increase the relative risk of metabolic syndrome, elevated fasting glucose levels, and high blood pressure. Additionally, moderate consumption of these drinks also increased the relative risk of elevated waist circumference in Brazilian adults (57). Similarly, soda and dietary fructose consumption were positively associated with a higher likelihood of hyperuricemia and elevated uric acid levels in Brazilian adults (58, 59).

Our findings underscore the importance of integrated public health strategies that address both physical activity and screen time to mitigate the consumption of unhealthy beverages. Additionally, considering that studies conducted within the ELSA-Brasil cohort showed that remaining physically active was associated with a 43% reduction in the risk of SARS-CoV-2 infection among those who also adopted specific COVID-19 protective practices (60), especially when combined with COVID-19 protective measures, public health interventions should promote active lifestyles and mindful eating habits, particularly during periods of social restriction. Given the significant health risks associated with high consumption of sugary drinks, policies aimed at reducing their consumption, such as taxation and public awareness campaigns, are crucial.

Among the limitations of the present study is its cross-sectional design, which precludes causal associations. To address this inherent limitation, we implemented statistical adjustments for key sociodemographic and lifestyle confounders, including age, income, smoking status, and alcohol consumption, using a hierarchical theoretical modeling approach. However, prospective cohort studies remain necessary to establish temporal relationships between physical activity, screen time behaviors, and beverage consumption patterns. Additionally, due to the restrictions and social distancing measures established during the supplementary COVID phase study in ELSA-Brasil during the pandemic, it was not possible to collect anthropometric data for the inclusion of body composition adjustment variables in the statistical models. To partially mitigate this constraint, we incorporated pre-pandemic data from ELSA-Brasil’s Wave 3 for core sociodemographic variables and included remote work status as a proxy for pandemic-specific behavioral modifications. Future investigations should integrate anthropometric data from the next ELSA-Brasil study waves to enhance model precision.

Moreover, no quantitative data on food and beverage consumption were collected to estimate the specific quantities consumed of each type of drink and food, and thus caloric intake adjustments were not possible, complicating deeper analyses on possible dose–response effects of physical activity and screen time behavior on beverage consumption. Recently, a diet quality index was developed and validated to compare dietary consumption data across different waves of ELSA-Brasil, including the COVID wave (61), which is a potential line of analysis to further investigate the findings of the present study regarding the effects of physical activity and screen time behavior on diet quality. Screen time was a variable implemented in the supplementary COVID study, so longitudinal analyses to assess changes over time in screen time behavior across different waves are not possible. Nevertheless, our median-based cutoff (≤6 vs. >6 h/day) for screen time demonstrated conceptual alignment with previous Brazilian adult population studies, enhancing cross-study comparability. The composite physical activity/screen time variable enabled examination of synergistic behavioral effects, representing a methodological advancement in this research domain.

Despite these limitations, the present study provides valuable information on the combined behaviors of physical activity and screen time and their relationship with sugary drink consumption in a cohort of Brazilian adults, given that most studies published to date on this topic focus on the child and adolescent population. Additionally, a separate analysis by beverage type was conducted, which is scarce in the current literature and allows for the identification of possible differentiated behaviors in beverage consumption habits. The findings highlight the need for public health strategies that promote active lifestyles and reduce screen time to mitigate the consumption of unhealthy beverages and their metabolic consequences. Moreover, the robust methodology and large sample size of the ELSA-Brasil study provide reliable data from a segment of the Brazilian population.

### 4.1 Practical applications and clinical relevance

The observed associations between the “Excessive Screen Time/Physically Inactive” behavioral pattern and beverage consumption patterns during the COVID-19 pandemic highlight the importance of monitoring these behavioral interactions in similar public health crises. Our findings, documented under specific pandemic conditions including social distancing and remote work, provide evidence to guide surveillance strategies during periods of prolonged confinement or restricted mobility.

In clinical practice, the results suggest that during extended periods of enforced sedentary behavior, routine assessment of both screen time and physical activity levels could serve as indicators for identifying adults at higher risk of unhealthy beverage consumption patterns, potentially informing Health and Food and Nutritional Education strategies. The sex-specific differences we observed in beverage consumption associations indicate that gender-specific monitoring approaches may be warranted when evaluating behavioral changes during lockdowns or restricted mobility periods. These findings contribute empirical evidence to understanding how sudden lifestyle disruptions may influence dietary behaviors in adult populations.

## 5 Conclusion

Significant differences were observed between genders regarding sociodemographic and lifestyle variables. Beverage consumption patterns varied significantly according to these variables. Participants who consumed soft drinks or soda, sugar-sweetened beverages, and coffee with sugar generally had lower per capita incomes, higher screen time, and were physically inactive. Conversely, those consuming coffee without sugar had higher per capita incomes and tended to have higher screen time and physical inactivity.

Excessive screen time combined with physical inactivity was significantly associated with higher odds of consuming sugar-sweetened artificial beverages among both men and women. Men with appropriate screen time but physically inactive behavior had nearly double the likelihood of consuming sugar-sweetened artificial beverages, while excessive screen time combined with physical inactivity significantly increased the likelihood of consuming these beverages among both men and women.

Additionally, excessive screen time and physical inactivity among men were associated with higher consumption of industrialized juices, whereas among women, this behavior increased the likelihood of consuming soft drinks or soda. No significant associations were found between physical activity and screen time behavior and the consumption of coffee, whether with or without sugar.

The demonstrated associations suggest public health strategies should prioritize integrated behavioral interventions that simultaneously target screen time reduction and physical activity promotion. Gender-specific approaches appear warranted, particularly programs addressing industrialized juice consumption in men and soda intake in women. For lower-income populations, where unhealthy beverage consumption was most prevalent, targeted nutritional support could help mitigate these patterns. The identified threshold of >6 h/day screen time combined with physical inactivity provides a clear benchmark for identifying high-risk groups needing intervention.

These findings offer evidence-based parameters for developing effective strategies to improve beverage consumption patterns. The study specifically highlights how addressing screen time and physical activity as interconnected behaviors, while considering gender and socioeconomic factors, could lead to more impactful public health interventions. The results provide measurable targets for program development and evaluation in community and clinical settings.

## Data Availability

The original contributions presented in the study are included in the article/Supplementary material, further inquiries can be directed to the corresponding author.
