# Correction: Physical activity and screen time behavior, and non-alcoholic beverage consumption during the COVID-19 pandemic in the longitudinal study of adult health (ELSA-Brasil)

**DOI:** 10.3389/fnut.2025.1674419

**Published:** 2025-08-18

**Authors:** Yazareni José Mercadante Urquía, Haysla Xavier Martins, Taísa Sabrina Silva Pereira, Letícia Batista de Azevedo, Luís Carlos Lopes Júnior, Maria Del Carmen Bisi Molina

**Affiliations:** ^1^Post-graduate Program in Collective Health, Universidade Federal do Espírito Santo, Vitória, Brazil; ^2^Department of Health Sciences, Universidad de las Américas Puebla, Puebla, Mexico; ^3^Post-graduate Program in Nutrition and Health, Universidade Federal do Espírito Santo, Vitória, Brazil; ^4^Post-graduate Program in Nutrition and Longevity, Universidade Federal de Alfenas, Alfenas, Brazil

**Keywords:** screen time (ST), physical activity, carbonated beverages, sugar-sweetened beverages (SSB), COVID-19, health behavior, lifestyle

In the published article, the author Maria Del Carmen Bisi Molina was assigned as the sole corresponding author. The correct corresponding authors are Maria Del Carmen Bisi Molina (mdcarmen2007@gmail.com) and Taísa Sabrina Silva Pereira (taisa.silva@udlap.mx).

In the published article, the funders *Fundação de Amparo à Pesquisa e Inovação do Esp*í*rito Santo* (FAPES) and *Universidad de las Américas Puebla* were omitted. The corrected **Funding** statement is below.

The author(s) declare that financial support was received for the research and/or publication of this article. This research was supported by the Ministry of Health (Science and Technology Department - DCIT) and the Ministry of Science, Technology, and Innovation in Brazil (Conselho Nacional de Desenvolvimento Científico e Tecnológico - CNPq), grant numbers 0106 0212.00 and 405551/2015-0 BA; 01 06 0300.00 and 405543/2015-8 ES; 0106 0278.00 and 405552/2015-7 MG; and 01 06 0071.00 and 405544/2015-4 RJ; 01 06 0010.00 and 405545/2015-0 RS; 01 06 0115.00 and 405547/2015-3 SP. Additionally, it was funded by the Coordenação de Aperfeiçoamento de Pessoal de Nível Superior (CAPES) through a doctoral scholarship awarded to the researcher YM, and by a Level 1C Research Productivity Fellowship from the CNPq awarded to the researcher MM. The publication of this article was funded by Fundação de Amparo à Pesquisa e Inovação do Espírito Santo (FAPES) and Universidad de las Américas Puebla.

The original version of this article has been updated.

