# Supplementary material for: Physical activity and screen time behavior, and non-alcoholic beverage consumption during the COVID-19 pandemic in the longitudinal study of adult health (ELSA-Brasil)
Source: Front Nutr. 2025 May 2;12:1503010. doi: 10.3389/fnut.2025.1503010 (PMC12083014; doi:10.3389/fnut.2025.1503010)
Supplement: Supplementary file 1 [file Data_Sheet_1.PDF]

Supplementary Material

1 Supplementary Figures and Tables

Supplementary Table 1. Sociodemographic Characteristics among Participants of the COVID Supplementary Study in ELSA-Brasil by Physical Activity and Screen Time Behavior (2020-2021).

Variables	Physical Activity Behavior				*p* value$_a$
	Appropriate ST/Physically Active n(%)	Active Appropriate ST/Physically Inactive n(%)	Excessive ST/Physically Active n(%)	Excessive ST/Physically Inactive n(%)	
**Gender**					
Male	233 (48.7)	791 (39.2)	191 (48.4)	642 (41.3)	**<.001***
Female	245 (51.3)	1225 (60.8)	204 (51.6)	911 (58.7)	
**Age (years)** (n=4357)					
	63.5±8.5 [a]	64.1±8.6 [a]	58.5±7.6 [b]	59.7±8.0 [b]	**<.001***
**Race** (n=4318)					
White	280 (60.1)	1044 (53.0)	244 (63.0)	909 (60.7)	**<.001***
Black, Brown, Yellow, and Indigenous	186 (39.9)	924 (47.0)	143 (37.0)	588 (39.3)	
***Per capita* income (U$)** (n=4346)					
	909,7±658,6 [a,c]	729,9±559,2 [b]	995,2±630,9 [a]	840,1±570,3 [c]	**<.001***
**Employment status** (n=4416)					
Employed/Working	225 (47.4)	893 (44.6)	321 (81.7)	1173 (76.0)	**<.001***
Retired	250 (52.6)	1111 (55.4)	72 (18.3)	371 (24.0)	
**Remote work** (n=2608)					
Yes	155 (69.2)	515 (57.9)	297 (92.5)	1055 (89.9)	**<.001***
No	69 (30.8)	375 (42.1)	24 (7.5)	118 (10.1)	
**Smoking**					
Non-smoker	323 (67.6)	1290 (64.0)	286 (72.4)	1010 (65.0)	**.007***

Former smoker	135 (28.2)	596 (29.6)	92 (23.3)	428 (27.6)	
Current	20 (4.2)	130 (6.4)	17 (4.3)	115 (7.4)	
**Alcoholic beverage consumption**					
Yes	304 (63.6)	1146 (56.8)	286 (72.4)	1020 (65.7)	**<.001***
No	174 (36.4)	870 (43.2)	109 (27.6)	533 (34.3)	

n=4442, Chi-square test was used for categorical variables and an ANOVA test for continuous variables. Different letters (a, b, c) indicate significant differences in Tukey's post hoc test.
* *p* values <0.05 were considered statistically significant.

Supplementary Table 2. Unadjusted and Adjusted Odds Ratios (95% CI) for Non-Alcoholic Beverage Consumption by Physical Activity and Screen Time Behavior in the ELSA-Brasil COVID-19 Supplementary Study (2020-2021).

**Variables**	**Unadjusted Model OR (95% CI)**	**Adjusted Model 1 OR (95% CI)**	**Adjusted Model 2 OR (95% CI)**
		**MALE**	
**Physical Activity and Screen Time Behavior**		**Soft drink or soda Consumption [c]**	
Appropriate ST/Physically Active	Reference	Reference	Reference
Appropriate ST/Physically Inactive	**1.62 (1.08-2.43)***	1.46 (0.85-2.52)	1.45 (0.84-2.50)
Excessive ST/Physically Active	0.83 (0.47-1.47)	0.82 (0.41-1.63)	0.81 (0.41-1.62)
Excessive ST/Physically Inactive	**1.82 (1.20-2.74)***	**1.75 (1.02-2.98)***	1.70 (0.99-2.91)
**Physical Activity and Screen Time Behavior**		**Sugar-sweetened artificial beverages [c]**	
Appropriate ST/Physically Active	Reference	Reference	Reference
Appropriate ST/Physically Inactive	**1.69 (1.22-2.35)***	**1.93 (1.22-3.06)***	**1.93 (1.22-3.06)***
Excessive ST/Physically Active	1.05 (0.68-1.62)	1.17 (0.67-2.03)	1.18 (0.68-2.05)
Excessive ST/Physically Inactive	**2.05 (1.47-2.86)***	**2.46 (1.56-3.87)***	**2.44 (1.54-3.84)***
**Physical Activity and Screen Time Behavior**		**Industrialized juices [c]**	
Appropriate ST/Physically Active	Reference	Reference	Reference
Appropriate ST/Physically Inactive	1.42 (0.94-2.14)	1.61 (0.92-2.84)	1.61 (0.91-2.84)
Excessive ST/Physically Active	1.17 (0.69-2.00)	1.23 (0.63-2.43)	1.24 (0.63-2.44)
Excessive ST/Physically Inactive	1.78 (1.18-2.69)	**2.04 (1.17-3.57)***	**2.02 (1.15-3.53)***
**Physical Activity and Screen Time Behavior**		**Coffee with sugar/sweetener Consumption**	
Appropriate ST/Physically Active	Reference	Reference	Reference
Appropriate ST/Physically Inactive	**1.56 (1.16-2.11)***	1.14 (0.75-1.73)	1.10 (0.72-1.67)

Excessive ST/Physically Active	**0.68 (0.46-0.99)***	0.67 (0.42-1.09)	0.66 (0.41-1.08)
Excessive ST/Physically Inactive	0.94 (0.70-1.28)	0.92 (0.62-1.38)	0.87 (0.58-1.31)
**Physical Activity and Screen Time Behavior**	**Coffee without sugar Consumption**		
Appropriate ST/Physically Active	Reference	Reference	Reference
Appropriate ST/Physically Inactive	**0.58 (0.44-0.80)***	**0.63 (0.40-0.98)***	0.65 (0.41-1.01)
Excessive ST/Physically Active	0.97 (0.64-1.46)	0.75 (0.45-1.24)	0.75 (0.45-1.26)
Excessive ST/Physically Inactive	0.92 (0.67-1.27)	0.81 (0.53-1.25)	0.85 (0.55-1.30)
	**FEMALE**		
**Physical Activity and Screen Time Behavior**	**Soft drink or soda Consumption** [c]		
Appropriate ST/Physically Active	Reference	Reference	Reference
Appropriate ST/Physically Inactive	**2.39 (1.42-4.00)***	**2.18 (1.05-4.52)***	1.95 (0.94-4.07)
Excessive ST/Physically Active	**2.22 (1.18-4.17)***	1.57 (0.68-3.61)	1.43 (0.62-3.31)
Excessive ST/Physically Inactive	**3.44 (2.05-5.78)***	**2.53 (1.23-5.23)***	**2.28 (1.10-4.72)***
**Physical Activity and Screen Time Behavior**	**Sugar-sweetened artificial beverages** [c]		
Appropriate ST/Physically Active	Reference	Reference	Reference
Appropriate ST/Physically Inactive	**1.66 (1.18-2.35)***	1.50 (0.88-2.55)	1.38 (0.81-2.36)
Excessive ST/Physically Active	**1.64 (1.05-2.55)***	1.37 (0.74-2.52)	1.27 (0.69-2.36)
Excessive ST/Physically Inactive	**2.11 (1.49-3.00)***	**1.86 (1.10-3.15)***	**1.72 (1.02-2.91)***
**Physical Activity and Screen Time Behavior**	**Industrialized juices** [c]		
Appropriate ST/Physically Active	Reference	Reference	Reference
Appropriate ST/Physically Inactive	1.19 (0.79-1.79)	0.97 (0.52-1.82)	0.94 (0.50-1.77)
Excessive ST/Physically Active	1.24 (0.72-2.12)	1.08 (0.52-2.22)	1.05 (0.51-2.16)
Excessive ST/Physically Inactive	1.31 (0.86-1.98)	1.12 (0.60-2.08)	1.09 (0.58-2.02)

Physical Activity and Screen Time Behavior	Coffee with sugar/sweetener Consumption		
Appropriate ST/Physically Active	Reference	Reference	Reference
Appropriate ST/Physically Inactive	**1.78 (1.35-2.35)***	**1.60 (1.02-2.53)***	1.50 (0.95-2.37)
Excessive ST/Physically Active	0.83 (0.57-1.20)	0.78 (0.46-1.33)	0.74 (0.44-1.27)
Excessive ST/Physically Inactive	1.18 (0.89-1.56)	1.04 (0.66-1.62)	0.98 (0.62-1.53)
**Physical Activity and Screen Time Behavior**	**Coffee without sugar Consumption**		
Appropriate ST/Physically Active	Reference	Reference	Reference
Appropriate ST/Physically Inactive	**0.56 (0.42-0.75)***	0.63 (0.38-1.04)	0.67 (0.41-1.11)
Excessive ST/Physically Active	1.28 (0.88-1.88)	1.39 (0.79-2.42)	1.42 (0.81-2.49)
Excessive ST/Physically Inactive	0.81 (0.60-1.09)	1.04 (0.64-1.68)	1.09 (0.67-1.76)

[a] Chi-square test for categorical variables and Student's t-test for continuous variables * *p* values <0.05 were considered statistically significant.
OR: *Odds Ratio*; **95% CI**: 95% Confidence Interval; *p* value <0.05; **Unadjusted Model:** Physical Activity and Screen Time Behavior; **Adjusted Model 1:** adjusted for Age (years); *Per Capita* Income (US$); Remote Work (Yes/No); **Adjusted Model 2:** Adjusted Model 1 + Smoking (Non-smoker, Former smoker, Current); Alcoholic beverage consumption (Yes/No); [c] Unadjusted for alcohol consumption.

1.1 Supplementary Figures

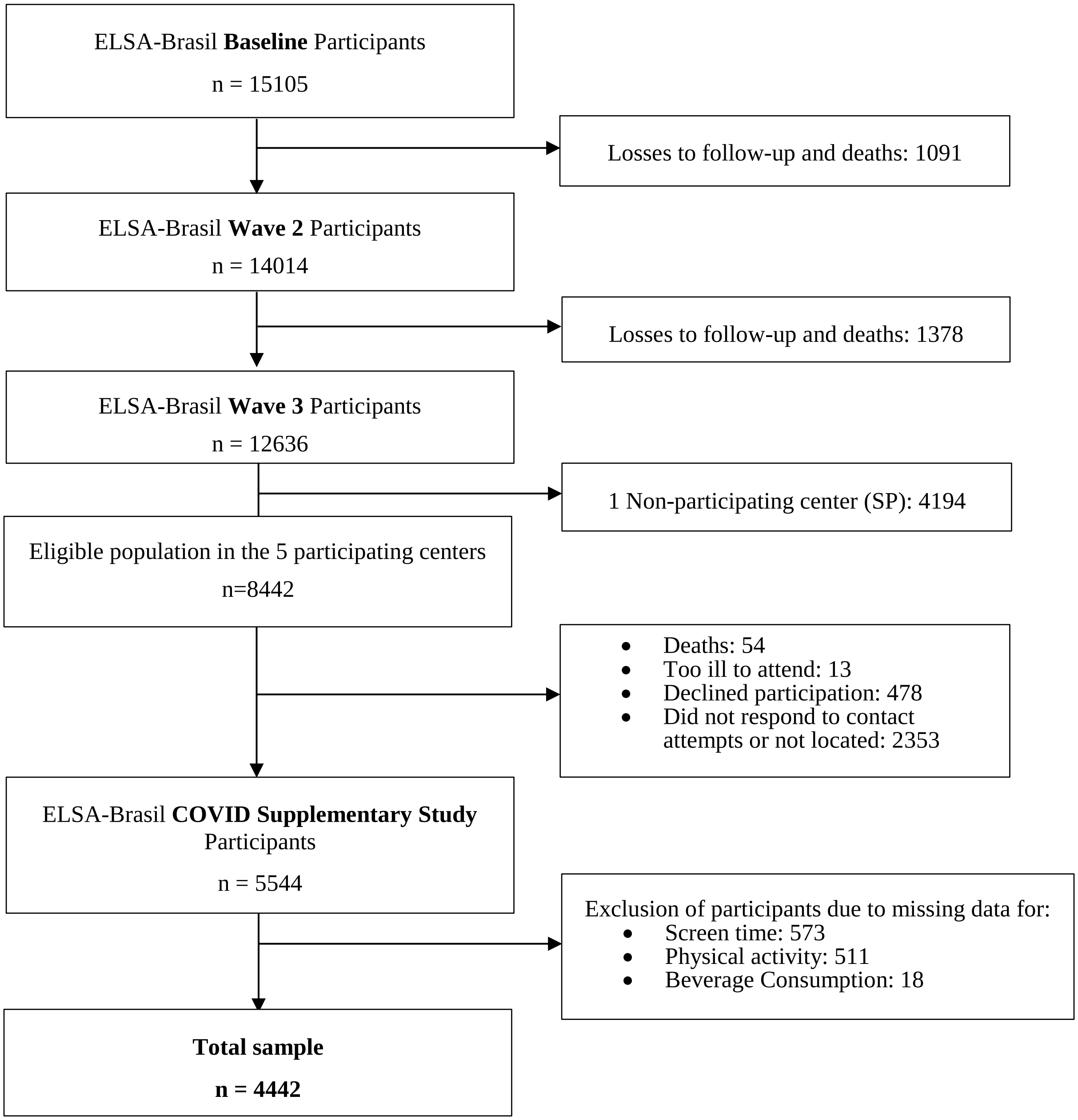

Supplementary Figure 1. Flowchart of Study Participants.
